# Temperature-Dependent Photoluminescence of CdS/ZnS Core/Shell Quantum Dots for Temperature Sensors

**DOI:** 10.3390/s22228993

**Published:** 2022-11-21

**Authors:** Luping Tang, Yangyang Zhang, Chen Liao, Yingqing Guo, Yingtao Lu, Yixuan Xia, Yiwei Liu

**Affiliations:** 1 College of Mechanical and Electrical Engineering, Nanjing Forestry University, Nanjing 210037, China; 2 SEU-FEI Nano-Pico Center, Key Lab of MEMS of Ministry of Education, Southeast University, Nanjing 210096, China; 3 College of Electronic and Optical Engineering & College of Flexible Electronics (Future Technology), Nanjing University of Posts and Telecommunications, Nanjing 210023, China

**Keywords:** temperature-dependent, photoluminescence, CdS/ZnS, quantum dots, temperature sensor

## Abstract

Exploring the temperature-dependent photoluminescence (PL) properties of quantum dots (QDs) is not only important for understanding the carrier recombination processes in QD-based devices but also critical for expanding their special applications at different temperatures. However, there is still no clear understanding of the optical properties of CdS/ZnS core/shell QDs as a function of temperature. Herein, the temperature-dependent PL spectra of CdS/ZnS core/shell QDs were studied in the temperature range of 77–297 K. It was found that the band-edge emission (BEE) intensity decreases continuously with increasing temperature, while the surface-state emission (SSE) intensity first increases and then decreases. For BEE intensity, in the low temperature range, a small activation energy (29.5 meV) in the nonradiative recombination process led to the decrease of PL intensity of CdS/ZnS core/shell QDs; and at high temperature the PL intensity attenuation was caused by the thermal escape process. On the other hand, the temperature-dependent variation trend of the SSE intensity was determined by the competition of the trapping process of the surface trap states and the effect of thermally activated non-radiative defects. As the temperature increased, the PL spectra showed a certain degree of redshift in the peak energies of both band-edge and surface states and the PL spectrum full width at half-maximum (FWHM) increases, which was mainly due to the coupling of exciton and acoustic phonon. Furthermore, the CIE chromaticity coordinates turned from (0.190, 0.102) to (0.302, 0.194), which changed dramatically with temperature. The results indicated that the CdS/ZnS core/shell QDs are expected to be applied in temperature sensors.

## 1. Introduction

The increasing demand for high-performance optoelectronic devices in recent years has attracted considerable attention to quantum dot (QD) systems, because QDs can confine their carriers in three dimensions [1,2]. QD devices have better performance than quantum well and quantum wire devices, such as high characteristic temperature, low threshold current density, and so on [3,4,5]. However, until now, QD devices have faced an important problem, which is that their performance degrades when they operate at high temperatures [6]. Temperature-dependent photoluminescence characteristics of QDs are very meaningful for QD-based devices, such as photovoltaic, light concentrators and phosphor conversion light emitting diodes (LEDs) [7,8]. These functional devices should be stable with high performance at elevated temperatures. Thus, it is very necessary to investigate the changes of optical properties of QDs with temperature. 

CdS QDs are an important material applied in photoelectric conversion and LED lighting [9,10]. The fluorescence efficiency of CdS QDs is relatively low, about 20–25%. To obtain more stable blue light emission, several layers of semiconductor materials with large band gaps, such as ZnS, are usually passivated on the surface of CdS QDs, increasing the coordination of surface atoms and blocking the interaction of excitons in CdS QDs with those in the surrounding environment, thus obtaining CdS/ZnS core/shell QDs with good photostability and high photoluminescence (PL) quantum yield [11,12]. At present, QD-LEDs prepared with CdS/ZnS core/shell QDs show highly efficient electroluminescence properties [13,14,15]. Further improvements in the performance of these QD-based devices depend not only on higher QD PL quantum yield, but also on better temperature stability. Some temperature-related studies have been carried out on the QDs of CdSe [16,17], CdTe [18], Ag_2_Se [19], PbSe [20,21], CuInS [22], indicating that the band gap and luminescence properties of semiconductors are temperature dependent. For instance, with the decrease of temperature, the electroluminescence intensity and efficiency of CdSe/ZnS core/shell QDs gradually increase, and the electroluminescence spectra show a significant blue shift [16]. The PL energy shift of CuInS_2_-ZnS core-shell QDs and CuInS_2_ QDs with temperature are 35 meV and 98 meV, respectively, which are much larger than those of the excitons in corresponding bulk semiconductors. However, there is still no clear understanding of the optical characteristics of CdS/ZnS core/shell QDs as a function of temperature [22].

Moreover, compared to traditional optical temperature probes such as organic fluorophores, luminescent complexes and inorganic phosphors, QDs have the advantages of multiparameter detectable signals, strong PL intensity, and narrow half-peak width when applied in optical temperature sensors. However, some difficult problems are still unsolved, including unsatisfactory response sensitivity, optional detectable signals, poor thermal stability, a limited temperature response range and so on [23,24]. Thus, designing a new optical temperature sensor based on QDs with multiparameter detectable signals, high sensitivity, and stability is still a challenge.

In this paper, the temperature-dependent PL spectra of CdS/ZnS core/shell QDs in the temperature range of 77–297 K were investigated. The decrease of PL intensity, the broadening of the PL spectrum, and the shift of emission peak energy changed with increasing temperature. It was found that the band-edge emission (BEE) decreased continuously, while the surface-state emission (SSE) did not seem to be a monotonic process with the increase of temperature. The PL spectra showed a certain degree of redshift in the peak energies of both band-edge and surface states with increasing temperature. Meanwhile, the PL linewidth increased with temperature, and was analyzed according to the standard equation describing the change of exciton width in the ground state with temperature. Furthermore, it was demonstrated that CdS/ZnS core/shell QDs as an optical temperature sensor possesses high sensitivity up to 2.14% K^−1^, and multiparameter detectable signals.

## 2. Materials and Methods

### 2.1. Synthesis of CdS/ZnS Core/Shell QDs

CdS/ZnS core/shell QDs were prepared based on a thermal cycling-SILAR method reported in our previous work [25]. First, CdS core QDs was synthesized. 4 g of oleic acid, 0.128 g of CdO and 35 mL of ODE were charged into a 250 mL three-necked flask and heated at 250 °C in an argon atmosphere. 5 mL of 0.1 M sulfur precursor solution was rapidly injected into the mixed solution, and the temperature was maintained at 230 °C for 30 min. Then, the obtained CdS core QDs were purified and transferred to a 250 mL three-necked flask, and 35 mL of ODE and 5ml of oleylamine were added. In an argon atmosphere, the temperature was raised to 240 °C, and 3.2 mL of Cd precursor solution was injected at the same time. After 10 min of growth, 3.2 mL of sulfur precursor solution was injected, and the growth was continued for 10 min, thus completing the preparation of a layer of ZnS shell layer. The precursor solution was injected according to the same method as above in the order of Zn-S-Zn-S-Zn-S-Zn-S. Finally, the synthesized core/shell QDs are purified by centrifugation, the excess organic ligands and unreacted substances are removed, and then the samples are dissolved in toluene.

### 2.2. Characterization

The absorption spectra of CdS/ZnS core/shell QDs were recorded with a Shimadzu UV-3600 spectrophotometer. 4 μL of the QD solution (n-hexane) was placed on the carbon film-coated copper grid and dried at room temperature to prepare transmission electron microscopy (TEM) samples, and the TEM images were all measured by FEI Tecnai G2 (200 kV). The diluted solution of the liquid-phase sample of CdS/ZnS core/shell QDs was deposited on a quartz plate. The quartz plate with the sample was fixed in a temperature holder of Oxford Company, and liquid nitrogen was used to cool down. The temperature characteristics of the samples were measured by the F900 steady-state/transient fluorescence spectrometer. The type of the QDs excitation laser was Edinburgh EPL-375 picosecond laser and the power density of the exciting laser radiation was 2 W/cm^2^.

## 3. Results and Discussion

**Figure 1:**
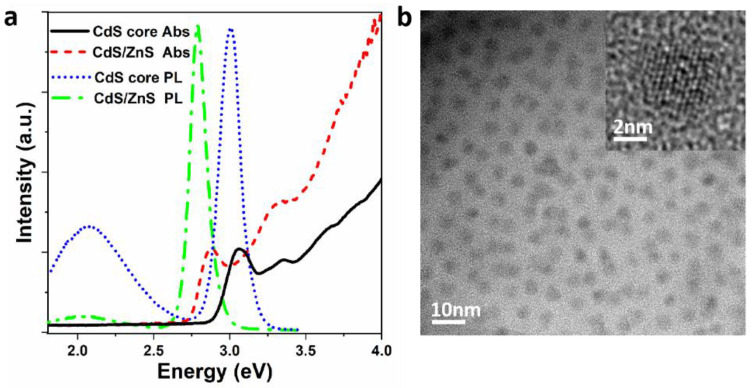
(**a**) Absorption and photoluminescence (PL) spectra of CdS core QDs (black solid line and blue dotted line) and CdS/ZnS core/shell QDs (red short dash line and green short dash dot line). (**b**) A typical transmission electron microscopy (TEM) image of CdS/ZnS core/shell QDs. Inset is corresponding high resolution TEM (HRTEM) image of the QDs.

Figure 1a displays the absorption and PL spectra of the CdS core and CdS/ZnS core/shell QDs at room temperature. It can be clearly seen that the position of the first exciton absorption peak of CdS core QDs (black solid line) and CdS/ZnS core/shell QDs (red short-dotted line) are 3.00 eV and 2.78 eV, respectively, displaying a certain degree of redshift. Similarly, corresponding position of the PL peak is also redshifted from 3.06 eV to 2.87 eV. The results show that with the increase of particle size, both the PL peak and the exciton absorption peak shift to higher wavelengths. At the same time, the long PL tail of CdS/ZnS core/shell QDs at the low energy side of the surface defect state is not as obvious as that of CdS core QDs, which also shows that the growth of the shell can effectively passivate the surface defect state. Figure 1b displays the TEM image of the CdS/ZnS core/shell QDs. According to the TEM image, the average size of the CdS/ZnS core/shell QDs is about 5.8 nm, which is significantly larger than the size of the CdS core QDs (~3.5 nm).

**Figure 2:**
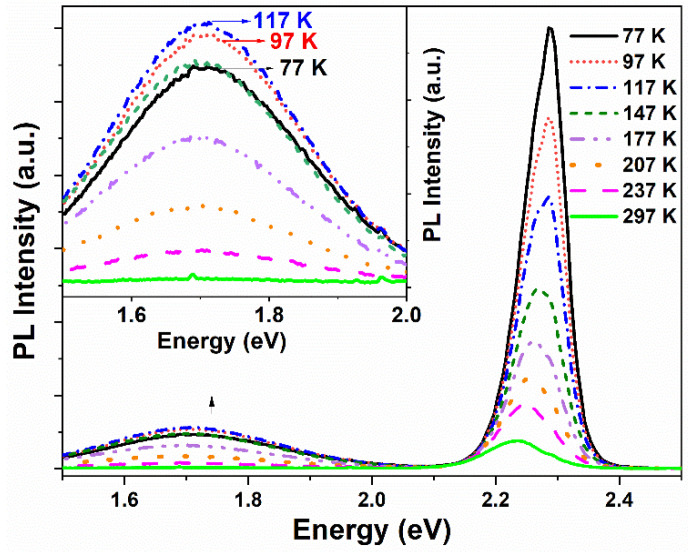
PL spectra of CdS/ZnS core/shell QDs at different temperatures from 77 to 297 K. Inset shows the magnified PL spectra of surface state of CdS/ZnS core/shell QDs.

Figure 2 displays the temperature-dependent PL spectra of CdS/ZnS core/shell QDs in the temperature range of 77–297 K, under excitation at 375 nm. Inset displays the magnified PL spectra of surface state of CdS/ZnS core/shell QDs. Obviously, as the temperature increased, the decrease of PL intensity, the broadening of the PL spectrum, and the shift of emission peak energy changed. As shown in Figure 2, the BEE intensity decreases continuously, while the SSE intensity does not seem to be a monotonic process with the increase of temperature. The PL spectra show a certain degree of redshift in the peak energies of both band-edge and surface states with increasing temperature. Moreover, the PL spectrum full width at half-maximum (FWHM) increases with the temperature. In order to deeply understand this change mechanism, the phonon energy and exciton phonon coupling are studied by comparing the experimental data with the fitting results, so as to obtain the internal interactions such as generation coupling and transition mechanism.

**Figure 3:**
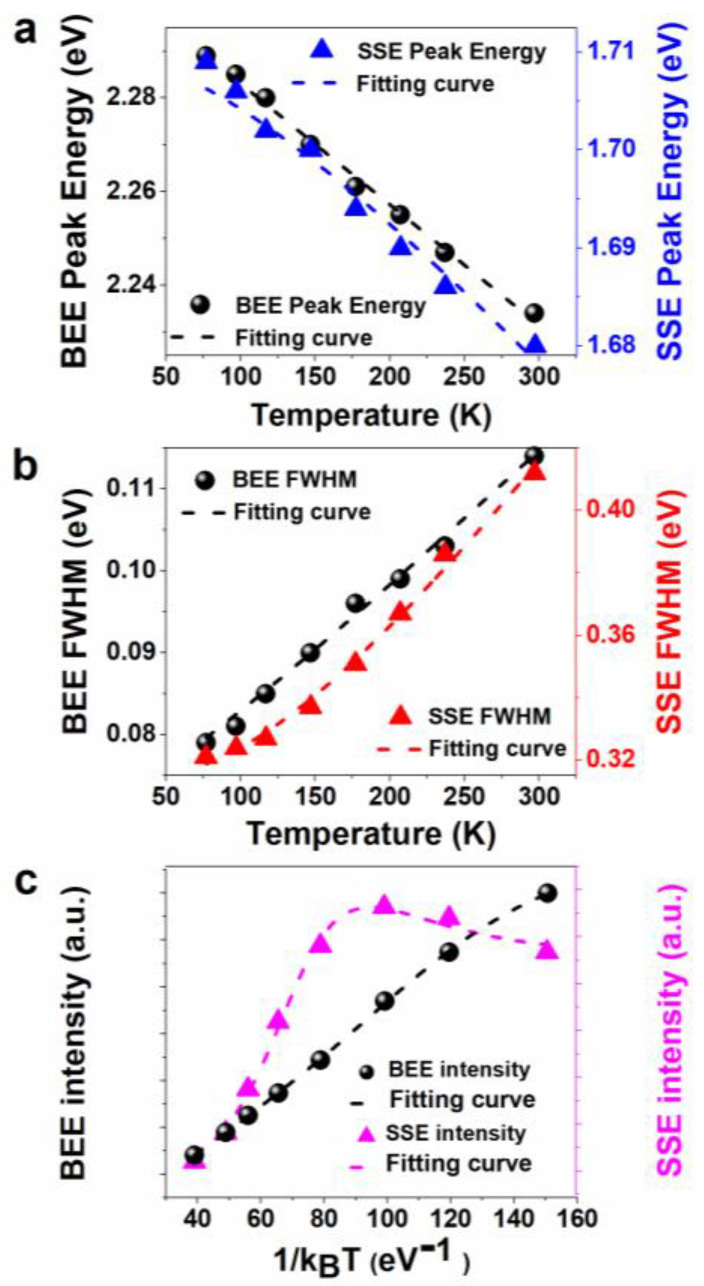
Temperature-dependent PL peak energy (**a**); and FWHM (**b**) of CdS/ZnS core/shell QDs in a temperature range of 77–297 K, respectively. Short dotted lines represent the fitting curves; (**c**) PL intensities of CdS/ZnS core/shell QDs as a function of inverse $k_{B}T$ in a temperature range of 77–297 K. In the figure, spheres represent BEE peak energy, BEE FWHM and BEE intensity; triangles represent SSE peak energy, SSE FWHM and SSE intensity.

In semiconductor nanostructures, except for the temperature-independent energy shift caused by quantum confinement, the temperature dependence of the energy gap is usually analogue to that of bulk semiconductor structures [26,27]. The redshift of semiconductor PL peak wavelength demonstrates that the energy band gap shrinks with the increase of temperature owing to exciton phonon coupling and lattice deformation [28,29]. The emission peaks of CdS/ZnS core/shell QDs varied with temperature (Figure 3a), which can be well fitted with the Varshni relationship [30]:

(1) $$E_{g}\left(T\right)=E_{g0}-\alpha \frac{T^{2}}{T+\beta }$$

$E_{g0}$ in the formula represents the energy gap at 0 K, α represents the temperature coefficient, β represents a fitting parameter, related to Debye temperature. The changes in lattice parameters and the temperature dependent electron lattice interaction are taken into account in this equation. Equation (1) is first proposed for infinite crystals; it can still be used to analyze bulk semiconductors and QDs. The α and β values of CdS/ZnS core/shell QDs are obtained to be 1.84 × 10^−4^ eV/K and 230 K, which are analogue to those for bulk CdS materials ($\alpha =3.3\times 10^{-4}\;\mathrm{eV}/K,\;\beta =221\;K$) [31,32]. This shows that the main emission in CdS/ZnS core/shell QDs comes from the recombination of holes and electrons near the band edge of CdS core. Considering the quantum confinement effect, the PL band movement of CdS/ZnS core/shell QDs with temperature is analogue to the temperature dependence of band gap contraction of bulk materials.

In addition, we can also observe the change of the PL spectral FWHM of CdS/ZnS core/shell QDs with temperature through Figure 3b, and the specific value can be acquired from the Gaussian best fit of the spectrum; it can be seen that the FWHM increase with the temperature. Part of PL broadening is uneven and part of it is homogeneous, which originates from exciton phonon scattering. Thus, it is of great significance to investigate the mechanism of spectral line broadening caused by exciton phonon scattering. Experimental data is fitted with the following equation [33,34], describing the temperature dependence of exciton peak broadening in bulk semiconductors and can be applied for CdS/ZnS core/shell QDs.

(2) $$\Gamma \left(T\right)=\Gamma_{inh}+\sigma T+\Gamma_{LO}{\left(e^{\frac{E_{LO}}{k_{B}T}}-1\right)}^{-1}$$

Here, $\Gamma_{inh}$ is a non-uniform broadening, independent of temperature, which is induced by fluctuations in the composition, shape, and size of nanocrystals; The latter two items are the uniform broadening caused by the exciton phonon interaction, σ represents the exciton acoustic phonon coupling coefficient, $\Gamma_{LO}$ is the exciton-Longitudinal optical (LO) phonon coupling coefficient, $E_{LO}$ is LO phonon energy, $k_{B}$ is Boltzmann constant. The temperature dependence of the FWHM energy for CdS/ZnS QDs can be well fitted, as displayed in Figure 3b. $E_{LO}$, is obtained to be 37 meV, which agreed with the value in a previous report [35]. The parameters $\Gamma_{inh}$ are calculated to 69 meV, and the smaller value shows that the particle size distribution is uniform, consistent with the TEM results. The parameters σ and $\Gamma_{LO}$ are determined to be 136.3 μeV/K and 15 meV, respectively. It shows that the exciton−acoustic phonon coupling has a great contribution to the spectral line broadening. Therefore, for CdS/ZnS core/shell QDs in a temperature range of 77–297 K, the broadening and displacement of the spectral line are mainly caused by the coupling of exciton and acoustic phonon.

**Figure 4:**
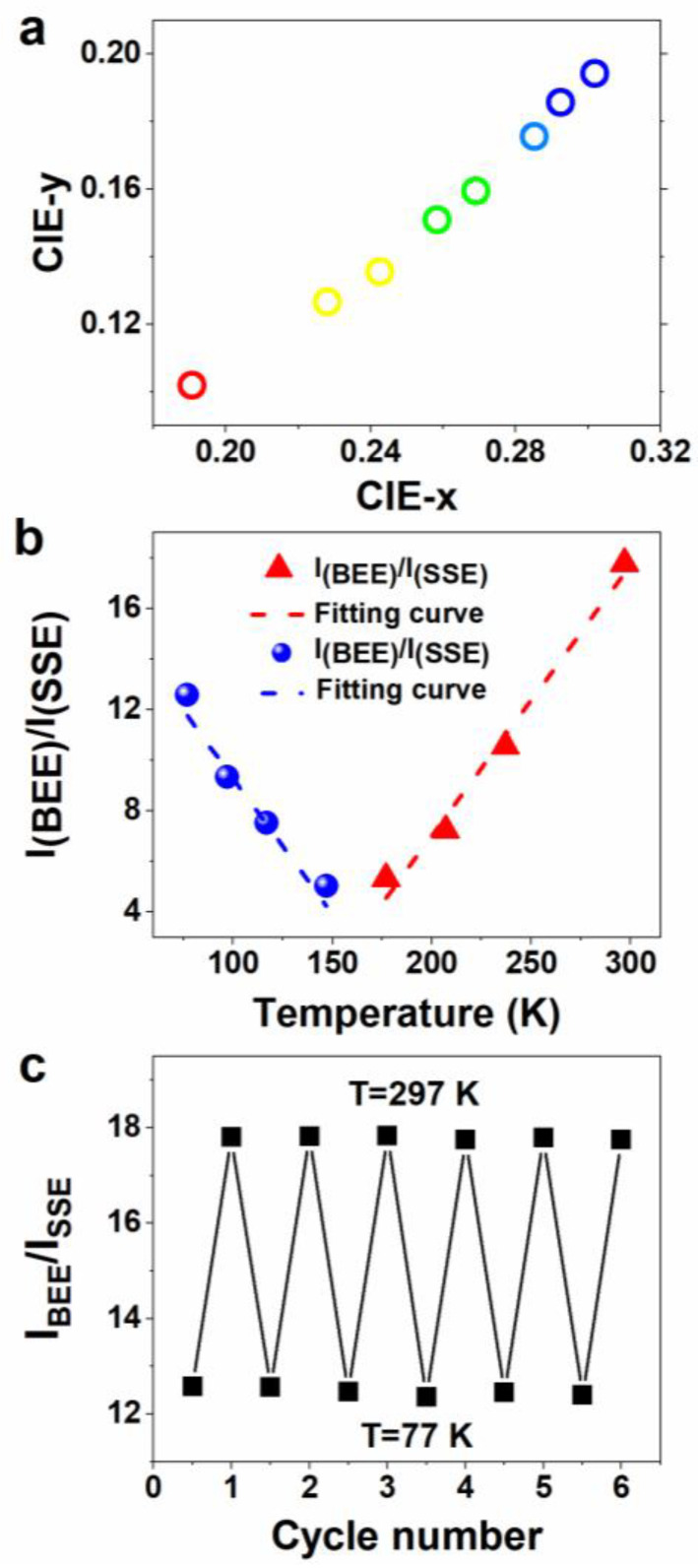
(**a**) Temperature-dependent emission from the CdS /ZnS core/shell QDs shown in Figure 2 projected onto the Commission Internationale del’Éclairage (CIE) chromaticity diagram; (**b**) ratio of integral PL peak areas of BEE and SSE; and (**c**) thermal stability of the integrated BEE and SSE PL peak area ratio over six cycles of heating and cooling between 77–297 K.

To investigate the role of different nonradiative processes in QD relaxation, the temperature dependence of the PL intensity was analyzed. The BEE intensity of CdS nanocrystals decreases as the temperature increases. The PL intensities of CdS/ZnS core/shell QDs as a function of inverse $k_{B}T$ is shown in Figure 4a. Considering two typical non radiative relaxation processes, including (1) carrier capture of surface defect states [36] and (2) multiple LO phonon assisted thermal escape [37,38], the temperature-dependent PL intensity can be fitted through the following relationship: [39,40]

(3) $$I_{PL}=\frac{I_{0}}{1+Ae^{\frac{-E_{a}}{k_{B}T}}+B{\left(e^{\frac{E_{LO}}{k_{B}T}}-1\right)}^{-m}}$$

where $I_{PL}$ represents the integrated PL intensity at different temperatures, $I_{0}$ represents the initial PL intensity at 0 K, $E_{a}$ represents activation energy of thermal quenching, $E_{LO}$ represents the average phonon energy, $k_{B}$ represents the Boltzmann constant, m represents the number of LO phonons involved in carrier thermal escape, and A and B are the ratios of the radiative lifetime to the capture time of nonradiative recombination centers, respectively. The black short dotted line in Figure 3c shows the simulation of BEE intensity exploiting this equation, which is very consistent with the experimental results, indicating that the decrease of BEE intensity can be explained by the carrier traps of surface defect states and the thermal escape assisted by multiple LO phonon scattering. The number of LO phonon m and the activation energy of carrier-trapping by surface defect states were obtained to be 3.3 and 29.5 meV, respectively. At the low temperature range, a small activation energy about 10–30 meV in the nonradiative relaxation process leads to the decrease of the PL intensity of CdS/ZnS core/shell QDs [37,41]. At high temperatures, the PL intensity attenuation is caused by the thermal escape process. It is worth noting that the SSE intensity of CdS/ZnS core/shell QDs first increases and then decreases with the increase of temperature. According to Equation (3) and the previous analysis on the change of BEE peak intensity with temperature, the carriers of the band edge will be transferred to the surface states, that is, the trapping process of the surface trap states, which will cause an increase in the surface-state luminescence intensity. At the same time, the effect of thermally activated non-radiative defects also exists in this luminescence process, so the temperature-dependent variation trend of the surface state luminescence intensity is determined by the competition of these two processes.

According to the analysis of a series of temperature changing phenomena of CdS/ZnS core/shell QDs, it was found that the temperature changing phenomena can be applied to the preparation of temperature sensors. As shown in Figure 4a, the CIE chromaticity diagram depicts the trajectory of (x, y) coordinates of the temperature-dependent PL spectra from the CdS/ZnS core/shell QDs shown in Figure 2. The CIE chromaticity coordinates varied dramatically from (0.190, 0.102) to (0.302, 0.194) with temperature, which also indicates its potential application in temperature sensors.

The corresponding temperature-induced BEE peak energy, BEE FWHM, BEE intensity shift (Figure S1a–c, black sphere symbols) can be described by a linear function (red solid line): $W=-2.5775\times 10^{-4}T+2.30881$, $W=1.58895\times 10^{-4}T+0.06644$, W=2581.16439T+80,115.15603, where T is the temperature. The linear fitting degree is expressed by Adj. R-Square, whose value is close to 1 the better. It can be seen from Figure S1 that the Adj. R-Square of the relationship between temperature and BEE peak energy, BEE FWHM, BEE intensity of the CdS/ZnS QDs sensor are all greater than 0.99, which means they all have obvious linear relationship.

The temperature-dependent dual-emission PL spectra of the CdS/ZnS QDs can also be characterized by ratio measurement. As shown in Figure 4b, the ratiometric measurement result of the integrated PL peak area, $R=I_{BEE}⁄I_{SSE}$, shows two different linear relationships. It displays a linear function relationship of R=−0.10743T+20.03117 at low temperatures (77−160 K) and R=0.10711T−14.39874 in the range of 160−297 K. By analyzing the data displayed in Figure 2, the maximum sensitivity value for the CdS/ZnS QDs is calculated to be 2.14% K^−1^ at 297 K. Here, thermal sensitivity is defined as S=1dR/RdT, where R is the integrated PL peak area ratio at a given temperature (T) [42,43]. It is worth noting that the thermal sensitivity is higher than most previously reported semiconductor QD based temperature sensors (Table S1). Therefore, $I_{BEE}⁄I_{SSE}$ can be used as a further parameter to obtain the precise temperature.

Since the BEE peak energy, BEE FWHM, BEE intensity and $I_{BEE}⁄I_{SSE}$ can be obtained by monitoring the PL spectra, the temperature value can be calculated using the corresponding linear fitting relationships. Furthermore, the cycling experiment results in Figure 4c exhibits very small changes in the $I_{BEE}⁄I_{SSE}$ during six consecutive cycles of repeatedly cooling and heating in the range of 77 to 297 K, indicating its excellent reliability and reversibility as a temperature sensor.

## 4. Conclusions

In summary, the temperature-dependent PL spectra of CdS/ZnS core/shell QDs were investigated in the temperature range of 77–297 K, and a series of temperature-changing phenomena were described and explained. It was found that the band-edge emission BEE intensity decreases continuously, while the SSE intensity first increases and then decreases with the increase of temperature. For BEE intensity, in the low-temperature range, a small activation energy (29.5 meV) in the nonradiative recombination process led to the decrease of PL intensity of CdS/ZnS core/shell QDs, and at high temperatures the PL intensity attenuation was caused by thermal escape process. The temperature-dependent variation trend of the SSE intensity is determined by the competition of the trapping process of the surface trap states and the effect of thermally activated non-radiative defects. Considering the quantum confinement effect, the redshift in the peak energies of both band-edge and surface states with increasing temperature is analogue to the temperature dependent band gap contraction of bulk materials. Moreover, the PL spectrum FWHM increased with the temperature, which mainly caused by the coupling of exciton and acoustic phonon. The CIE chromaticity coordinates changed dramatically from (0.190, 0.102) to (0.302, 0.194), with temperature, which also suggested that the CdS/ZnS core/shell QDs are expected to be applied in temperature sensors.

## Data Availability

Not Applicable.

## Supplementary Materials

The following supporting information can be downloaded at: https://www.mdpi.com/article/10.3390/s22228993/s1, Figure S1: Temperature-dependent band-edge emission (BEE) peak energy, BEE full width at half-maximum (FWHM) and BEE intensity of CdS/ZnS QDs, and corresponding linear fitting relation curve; Table S1: Comparison of previously reported semiconductor quantum dots (QDs)-based temperature sensors. References [24,42,44,45,46,47,48,49,50,51,52] are cited in the supplementary materials.
