# Supplementary material for: Temperature-Dependent Photoluminescence of CdS/ZnS Core/Shell Quantum Dots for Temperature Sensors
Source: Sensors (Basel). 2022 Nov 21;22(22):8993. doi: 10.3390/s22228993 (PMC9698013; doi:10.3390/s22228993)
Supplement: Supplementary file 1 [file sensors-22-08993-s001.zip › sensors-1998382-supplementary.pdf]

Supplementary Information

# Temperature-Dependent Photoluminescence of CdS/ZnS Core/Shell Quantum Dots for Temperature Sensors

**Luping Tang [1,2,*], Yangyang Zhang [1], Chen Liao [3], Yingqing Guo [1], Yingtao Lu [1], Yixuan Xia [1] and Yiwei Liu [1]**

[1] College of Mechanical and Electrical Engineering, Nanjing Forestry University, Nanjing 210037, China
[2] SEU-FEI Nano-Pico Center, Key Lab of MEMS of Ministry of Education, Southeast University, Nanjing 210096, China
[3] College of Electronic and Optical Engineering & College of Flexible Electronics (Future Technology), Nanjing University of Posts and Telecommunications, Nanjing 210023, China
* Correspondence: tlp@njfu.edu.cn

## List of SI

**Figure S1.** Temperature-dependent band-edge emission (BEE) peak energy, BEE full width at half-maximum (FWHM) and BEE intensity of CdS/ZnS QDs, and corresponding linear fitting relation curve.

**Table S1.** Comparison of previously reported semiconductor quantum dots (QDs)-based temperature sensors.

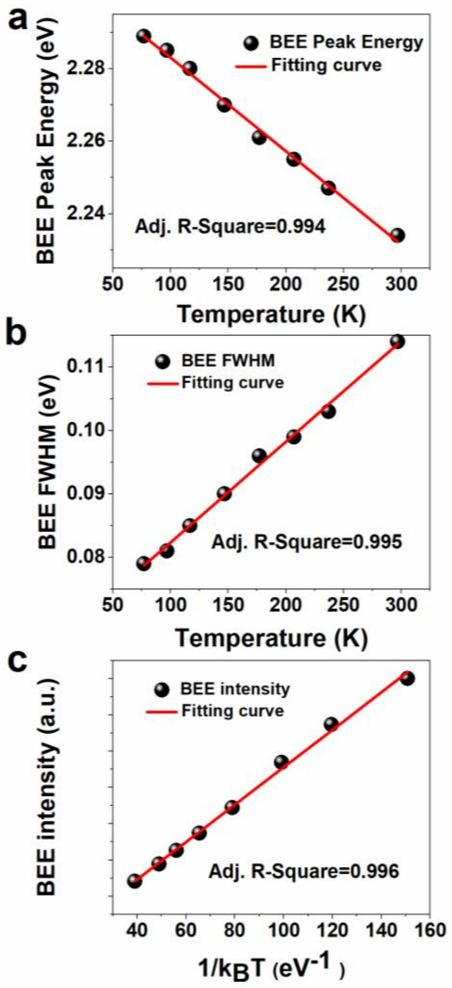


**Figure S1.** Temperature-dependent BEE peak energy (a) and BEE FWHM (b) of CdS/ZnS core/shell QDs in a temperature range of 77–297 K, respectively. (c) BEE intensity of CdS/ZnS core/shell QDs as a function of inverse $k_BT$ in a temperature range of 77–297 K.

**Table S1.** Comparison of previously reported semiconductor quantum dots (QDs)-based temperature sensors.

| Sensing materials | Sensitivity (% $K^{-1}$) | Temperature range (K) | Ref. |
|---|---|---|---|
| **CdS/ZnS** | 2.14 | 77−297 | In this work |
| **$Mn^{2+}$-doped CdS-ZnS QDs** | 0.5 | 77−320 | [1] |
| **CdSe/ZnS QDs** | 1.3 | 100−315 | [2] |
| **CdSe QDs** | 0.69 | 82−280 | [3] |
| **PbS/CdS/CdSe QDs** | 1.22 | 180−300 | [4] |
| **PbS/CdS giant QDs** | 1.13 | 150−373 | [5] |
| **$Mn^{2+}$-doped ZnSe-ZnCdSe QDs** | 0.9 | 82−280 | [6] |
| **$CuInS_2$ QDs** | 1.0 | 140−340 | [7] |
| **$Zn_{1-x}Mn_xSe$/ZnS/CdS/ZnS QDs** | 0.7 | 373−673 | [8] |
| **CdTe QDs film** | 0.83 | 23−80 | [9] |
| **CdTe QDs solution** | 0.57 | 23−80 | [9] |
| **CdTe QDs-Layered Double Hydroxides ultrathin films** | 1.47 | 23−80 | [9] |
| **CdTe QDs@NaCl** | 0.61 | 80−360 | [10] |
| **CdHgTe QDs@NaCl** | 1.4 | 80−340 | [11] |

**Reference**


1. Park, Y.; Koo, C.; Chen, H. Y.; Han, A.; Son, D. H. Ratiometric temperature imaging using environment-insensitive luminescence of Mn-doped core-shell nanocrystals. *Nanoscale* **2013**, *5*, 4944−4950.
2. Walker, G. W.; Sundar, V. C.; Rudzinski, C. M.; Wun, A. W.; Bawendi, M. G.; Nocera, D. G. Quantum-dot optical temperature probes. *Appl. Phys. Lett.* **2003**, *83*, 3555−3557.
3. Jethi, L.; Krause, M. M.; Kambhampati, P. Toward ratiometric nanothermometry via intrinsic dual emission from semiconductor nanocrystals. *J. Phys. Chem. Lett.* **2015**, *6*, 718−721.
4. Liu, J.; Zhang, H.; Selopal, G. S.; Sun, S.; Zhao, H.; Rosei, F. Visible and near-infrared, multiparametric, ultrasensitive nanothermometer based on dual-Emission colloidal quantum dots. *ACS Photonics* **2019**, *6*, 2479–2486.
5. Zhao, H. G.; Vomiero, A.; Rosei, F. Ultrasensitive, biocompatible, self-calibrating, multiparametric temperature sensors. *Small* **2015**, *11*, 5741−5746.
6. Vlaskin, V. A.; Janssen, N.; van Rijssel, J.; Beaulac, R.; Gamelin, D. R. Tunable dual emission in doped semiconductor nanocrystals. *Nano Lett.* **2010**, *10*, 3670−3674.
7. Marin, R.; Vivian, A.; Skripka, A.; Migliori, A.; Morandi, V.; Enrichi, F.; Vetrone, F.; Ceroni, P.; Aprile, C.; Canton, P. Mercaptosilane-passivated $CuInS_2$ quantum dots for luminescence thermometry and luminescent labels. *ACS Appl. Nano Mater.* **2019**, *2*, 2426−2436.
8. McLaurin, E. J.; Vlaskin, V. A.; Gamelin, D. R. Water-soluble dual-emitting nanocrystals for ratiometric optical thermometry. *J. Am. Chem. Soc.* **2011**, *133*, 14978−14980.
9. Liang, R.; Tian, R.; Shi; W.; Liu, Z.; Yan, D.; Wei, M.; Evans, D. G.; Duan, Xue. A temperature sensor based on CdTe quantum dots-layered double hydroxide ultrathin films via layer-by-layer assembly. *Chem. Commun.* **2013**, *49*, 969–971.
10. Kalytchuk, S.; Zhovtiuk, O.; Kershaw, S. V.; Zboril, R.; Rogach, A. L. Temperature-dependent exciton and trap-related photoluminescence of CdTe quantum dots embedded in a NaCl matrix: Implication in thermometry. *small* **2016**, *12*, 466–476.
11. Kalytchuk, S.; Adam, M.; Tomanec, O.; Zboril, R.; Gaponik, N.; Rogach, A. L. Sodium chloride protected CdHgTe quantum dot based solid-state near-infrared luminophore for light-Emitting devices and luminescence thermometry. *ACS Photonics* **2017**, *4*, 1459−1465.
